# A new protein curbs the hypertrophic effect of myostatin inhibition, adding remarkable endurance to motor performance in mice

**DOI:** 10.1371/journal.pone.0228653

**Published:** 2020-03-11

**Authors:** Marina Boido, Olena Butenko, Consuelo Filippo, Roberta Schellino, Jan W. Vrijbloed, Ruggero G. Fariello, Alessandro Vercelli

**Affiliations:** 1 Neuroscience Institute Cavalieri Ottolenghi, Department of Neuroscience "Rita Levi Montalcini", University of Torino, Torino, Italy; 2 PharmaFox Therapeutics AG, Möhlin, Aargau, Switzerland; University of Sydney, AUSTRALIA

## Abstract

Current efforts to improve muscle performance are focused on muscle trophism via inhibition of the myostatin pathway: however they have been unsuccessful in the clinic to date. In this study, a novel protein has been created by combining the soluble activin receptor, a strong myostatin inhibitor, to the C-terminal agrin nLG3 domain (ActR-Fc-nLG3) involved in the development and maintenance of neuromuscular junctions. Both domains are connected via the constant region of an Igg1 monoclonal antibody. Surprisingly, young male mice treated with ActR-Fc-nLG3 showed a remarkably increased endurance in the rotarod test, significantly longer than the single domain compounds ActR-Fc and Fc-nLG3 treated animals. This increase in endurance was accompanied by only a moderate increase in body weights and wet muscle weights of ActR-Fc-nLG3 treated animals and were lower than expected. The myostatin inhibitor ActR-Fc induced, as expected, a highly significant increase in body and muscle weights compared to control animals and ActR-Fc-nLG3 treated animals. Moreover, the prolonged endurance effect was not observed when ActR-Fc and Fc-nLG3 were dosed simultaneously as a mixture and the body and muscle weights of these animals were very similar to ActR-Fc treated animals, indicating that both domains need to be on one molecule. Muscle morphology induced by ActR-Fc-nLG3 did not appear to be changed however, close examination of the neuromuscular junction showed significantly increased acetylcholine receptor surface area for ActR-Fc-nLG3 treated animals compared to controls. This result is consistent with published observations that endurance training in rats increased acetylcholine receptor quantity at neuromuscular junctions and provide evidence that improving nerve-muscle interaction could be an important factor for sustaining long term muscle activity.

## Introduction

Optimal functioning of the muscle tissue depends on the correct interaction of several factors, two of which are pivotal: on one hand the balance between protein synthesis and degradation within the muscle fiber, and on the other hand the nerve activity through muscle innervation, both of which have electrical and trophic influences.

The correct balance of muscle protein metabolism is regulated by follistatin and myostatin [1]. Follistatin promotes protein synthesis and increases muscle mass mostly, but not only, by preventing myostatin binding to its receptor [2, 3]. Myostatin, after binding to the activin receptor 2B (ActR-IIB), initiates a cascade of reactions that eventually restricts growth of muscle mass [3]. The role of these two proteins has been extensively studied and their effects confirmed by multiple *in vitro* and *in vivo* experiments both in transgenic and natural animal models [4–9].

Skeletal muscle tissue is innervated by the second order motor neurons (MNs) located in the anterior horns of the spinal cord. Each MN innervates a variable number of muscle fibers, forming the motor unit, where muscles which need a finer control of movement have smaller motor units [10]. The nerve terminals form a highly specialized synapse called neuromuscular junction (NMJ) that not only sends signals for contraction, but also secretes and endocytoses important trophic factors in absence of which the muscle fibers undergo atrophy and degenerate [11]. Agrin is a large extracellular proteoglycan protein containing a large number of different domains but crucial for the functioning of the NMJ is the neuronal form of the C-terminal domain (nLG3) [12].

As a consequence of the above, optimal muscle performance relies on the synergistic action of complex biochemical mechanisms intrinsic to the muscle tissue, in addition to proper neurogenic inputs. Derangements of each step of these complex mechanisms result in impaired muscle performance in one, or all of its various aspects (strength, rapidity of execution and endurance, i.e. the capability of sustaining prolonged effort). Under normal circumstances, muscle performance reaches its peak in the third decade of life in humans [13] and declines with aging even in absence of disease [14]. Numerous approaches to strengthen muscle performance aimed at both reaching extreme performances and/or correcting pathological conditions. In the past few years, the main focus was on the manipulation of the myostatin system, either by inactivating myostatin with specific antibodies, blocking its receptor with specific antibodies or by using the soluble myostatin receptor as a decoy [15–20]. These strategies have resulted in substantial enlargement of muscle masses, in moderately improved muscle strength, but failed to increase endurance [21, 22].

We hypothesized that increasing muscle mass without adequate incremental innervation may, in the long run, be counterproductive to sustain prolonged physical activity. Thus, we designed and produced a new protein to improve both the myogenic and neurogenic component of muscle performance. This protein (ActR-Fc-nLG3) was administered during several weeks to normal young mice resulting in a moderate increase in muscle mass and strength and in a remarkable endurance in the rotarod test compared to PBS-treated controls and mice that received myostatin inhibitors only.

## Materials and methods

### Synthesis of proteins

All proteins and antibodies described were produced and purified by Evitria AG (Schlieren, Switzerland) and summarized in Table 1. The protein ActR-Fc [23] has been described before. Briefly, ActR-Fc consists of the mouse extracellular part of the ActR-IIB receptor coupled to the Fc part of an Igg1 mAb [23]. Fc-nLG3 refers to the neuronal laminin G3 domain of agrin [12, 24] coupled to the c-terminus of the Fc part of an Igg1 mAb.

**Table 1:** List of compounds used in this study.

Compound name	Comment, short description	Reference
**(m)ActR-Fc**	Mouse extracellular ActR-IIB receptor coupled to Fc constant region of an antibody	[23]
**ActR-Fc**	Human extracellular ActR-IIB receptor coupled to Fc constant region of an antibody (Ramatercept)	[23]
**(m)Fc-nLG3**	nLG3 domain of agrin coupled to the Fc constant region of an antibody	[12], this paper
**Fc-nLG3**	Human nLG3 domain coupled to the Fc constant region of an antibody	[12], this paper
**(m)ActR-Fc-nLG3**	ActR-Fc compound (see above) coupled to the nLG3 domain of agrin	This paper
**ActR-Fc-nLG3**	Human ActR-Fc compound (see above) coupled to the human nLG3 domain of agrin	This paper
**ActR-Fc-LG3**	Human ActR-Fc compound (see above) coupled to the human LG3 domain of agrin	This paper

### SDS-polyacrylamide gel electrophoresis

In order to verify the molecular weights of the synthetized compounds, each one was eluted in 4X LDS Sample Buffer (Invitrogen) and 10X reducing agent (Invitrogen) to reach the concentration of 1μg. Samples were heated at 70°C for 10 minutes, then were run on 4–12% Bis-Tris Plus gel (Invitrogen). Each chamber of the tank was filled with 400 mL of 1X SDS Running Buffer (obtained by mix 20 mL of 20X Invitrogen MES SDS Running Buffer with 380 mL of deionized water). Gels were run at 200V voltage for 35 minutes.

Target protein fractions were identified by Coomassie staining of gel. Gels were left in Coomassie staining solution (0.26% Coomassie Blue, 10% Acetic Acid, 25% Methanol) for 4 hours. After removing Coomassie solutions, gels were then incubated in the destaining solution (10% Acetic Acid, 25% Methanol) overnight, in order to eliminate the color in excess. Gels were then scanned and images were acquired using a densitometer (BioRad).

### *In vitro* experiments: AChR clustering

C2C12 mouse skeletal myoblasts from ATCC (ATCC-LGC Standards S.r.l., Italy) were cultured in Dulbecco’s Modified Eagle’s Medium (DMEM) high glucose (Sigma, St. Louis, MO, USA) with 10% Fetal Bovine Serum (FBS; Sigma), containing 2 mM L-glutamine, 100 U/ml penicillin and 100 μg/ml streptomycin (all purchased from Invitrogen-Gibco, Milan, Italy). The cells were cultured for 2–3 days on 8 well chamber slides in the previous medium, then replaced with DMEM and 3% FBS, to obtain myotubes. Then the cells were incubated with Fc-nLG3, ActR-Fc, ActR-Fc-nLG3 or ActR-Fc-LG3 at 10 μM for 24h and fixed with 2% paraformaldehyde for 20 min at RT. In addition, PBS was used as negative control. The samples were stained for the Acetylcholine receptor (AChR) by incubating the cells with Alexafluor 555-conjugated α-Bungarotoxin (1:500; Invitrogen, Italy) at RT for 1 h. The cells were rinsed and coverslips were mounted with a drop of PB 0.1M. The samples were then observed with a Leica TCS SP5 confocal laser scanning microscope (Leica Microsystems) and representative images acquired.

### Animal care and use

We used young C57BL/6j male mice, purchased from Harlan (Italy). All experimental procedures on live animals were carried out in strict accordance to the European Communities Council Directive 86/609/EEC (November 24, 1986) Italian Ministry of Health and University of Turin institutional guidelines on animal welfare (law 116/92 on Care and Protection of living animals undergoing experimental or other scientific procedures; authorization number 17/2010-B, June 30, 2010): additionally an ad hoc Ethical Committee of the University of Turin approved the study. All efforts were made to minimize the number of animals used and their suffering.

Animals were housed into ventilated cages in groups of four to five animals per cage, with a 12:12 h light-dark cycle. They were fed a standard laboratory diet containing 22% protein and 3.5% fat (Mucedola, Settimo Mil.se, Italy). Food and water were provided ad libitum.

### *In vivo* testing of nLG3 and ActR constructs

Nine-week-old male C57BL/6j mice (n = 5 per group) were randomized with body weight and treated subcutaneously (10 mg/kg) with the following proteins (murine version): (m)ActR-Fc-nLG3, (m)Fc-nLG3, (m)ActR-Fc, and a mixture of (m)ActR-Fc and (m)Fc-nLG3, three times per week. Controls received PBS, pH 7.4. The dose and dose frequency was selected based on the dose of ActR-Fc administrated in a previous study [23].

### Behavioral tests

Forelimb grip strength was measured using a Grip Strength Meter (Ugo Basile, Varese, Italy): mice were held by the tail and allowed to grasp a T-shaped bar with their forepaws. Once the mouse grasped the bar with both paws, the mouse was pulled away from the bar until the mouse released the bar. The digital meter displays the level of tension (in gram-force; gF) exerted on the bar by the mouse. Each animal was given five consecutive tests, and the average value was taken.

Rotarod measurements were done on a 7650-accelerating model of a rotarod apparatus (Ugo Basile, Comerio, VA, Italy). The mice were placed on the rod of a Rotarod, which was slowly accelerated from 4 to 32 rpm. Each animal underwent three trials, with the arbitrary cut-off time of 300 seconds (the best result of each trial has been considered). An additional extended trial was performed with a maximal duration of 2000 seconds (30 minutes). The first trials were considered as training, and then the performances on day 21 and 25 were formally registered.

### Histological analysis

At the end of the observation period, the mice were sacrificed by cervical dislocation. Gastrocnemius, quadriceps femoris and triceps brachii were collected, weighed and histologically analyzed.

ActR-Fc-nLG3 and PBS gastrocnemius muscles were then frozen using 2-methylbutane cooled with liquid nitrogen to preserve optimal skeletal muscle morphology. Muscles were then embedded in cryostat medium (Killik; Bio-Optica, Milan, Italy), cut on the cryostat (HM 550; Microm) in transverse 20 μm-thick sections and mounted onto 5% gelatin-coated slides: the sections were then stained with hematoxylin/eosin, dehydrated in graded ethanol baths (95–100%) and cleared in xylene. Reconstructions and analysis of the sections have been performed by Neurolucida software and the associated data analysis software NeuroExplorer (MicroBrightField). We evaluated mean fiber perimeter, maximum and minimum Feret diameters of fibers, gastrocnemius cross-sectional perimeter.

In addition, longitudinal muscle slices [PBS, (m)ActR-Fc, (m)ActR-Fc-nLG3, (m)ActR-Fc-LG3] were also collected and stained for the acetylcholine (ACh) receptor by incubating the tissues with Alexafluor 555-conjugated α-Bungarotoxin (1:500; Invitrogen, Italy) at RT for 30 min. The slides were then rinsed, coverslipped with the anti-fade mounting medium Mowiol and analyzed with a Leica TCS SP5 confocal laser scanning microscope (Leica Microsystems), using these parameters: objective 40x, format 1024x1024, scan speed 200 Hz, step size 0.49μm (until covering the whole NMJ volume), zoom 4.0, laser power 100%, gain range (V) 820–930, offset range (%) -20-23. Area and perimeter of the NMJs of 4 animals per group were analyzed by using ImageJ software (MIT). The wand tracing tool was used to select the α-Bungarotoxin stained area(s) of the NMJ. Subsequently the measured area and perimeter were determined with the “measure” function. Areas and perimeters of fragmented NMJs were added together to calculate the total area and perimeter of the NMJ. Additionally, as densitometric analysis, NMJ fluorescent signal was measured by Scion Image software for Windows (freeware version of NIH Image, Scion Corporation, Frederick, MD, USA): the background was subtracted from photographs by automatic thresholding [25, 26].

### Neuronal LG3 (nLG3) versus LG3

In another set of experiments, twenty 6-week-old C57BL/6j male mice were split randomly into four experimental groups (n = 5 per group). Control group received injections of PBS, whereas the other groups were treated with the following proteins (human version): ActR-Fc, ActR-Fc-nLG3 and ActR-Fc-LG3. Injections (10 mg/kg) were performed subcutaneously 3 times per week for three weeks. Body weight (BW) was measured 5 times per week during experiment.

Then mice were sacrificed by cervical dislocation, the fresh skeletal muscles (triceps, quadriceps and gastrocnemius) were quickly dissected and the wet muscle weight determined, as previously described.

### Statistical analysis

Data are presented as mean ± S.E.M. (standard error of mean). All the raw data are reported in S1 Dataset. Student’s unpaired t-test was used to determine significant differences in the morphological analysis of muscles (muscle perimeter, mean fiber length, maximum and minimum Feret diameters; ratio nuclei/100 fibers). One-way ANOVA was employed to analyze the weight increase (%) and the wet weight of muscles, and NMJ area, perimeter and fluorescent signal. Two-way ANOVA repeated measures was used to evaluate the wet weight of muscles (experiments with neuronal agrin), the BW increase and motor performance (rotarod) during time. Values of P<0.05 were considered significant (*), P<0.01 very significant (**) and P<0.001 extremely significant (***). The investigators were blind during the study and the analysis of the results.

## Results

### Assessment of the molecular weight of murine and human compounds

We have synthesized a compound that combines muscular hypertrophy (ActR, extracellular domain of the activin receptor) with neuromuscular innervation (nLG3, agrin). The ActR and nLG3 domains were fused to the Fc domain of an Igg1 antibody and the resulting compound is called actR-Fc-nLG3. In addition, control compounds actR-Fc, Fc-nLG3 and actR-Fc-LG3 were synthesized, to examine the effects of the individual effector domains. First of all, we verified the relative molecular mass of each synthetized compound (mouse- and human-derivatives) by SDS-polyacrylamide gel electrophoresis (PAGE), after Coomassie staining (S1 Fig). The results show that the molecular weights of mouse and human Fc-nLG3 and ActR-Fc are very similar, about 55 kDa. The molecular weight of mouse and human ActR-Fc-nLG3 is about 75 kDa. As expected, human ActR-Fc-LG3 is nearly identical to ActR-Fc-nLG3: indeed ActR-Fc-LG3 differs for only 8 amino acids compared to ActR-Fc-nLG3.

### *In vivo* testing of nLG3 and ActR constructs

With the aim to verify the ability of (m)ActR-Fc-nLG3 to simultaneously promote both muscle hypertrophy and innervation, we administrated this compound to nine-week-old mice. At this age, the development of NMJs and muscles is completed [27]. The average starting weight of the animals was 28.55 ± 0.5 g and 29.21 ± 0.4 g respectively for the controls (PBS-treated) and the (m)ActR-Fc-nLG3 animals. Since the mice were young, the BWs of both the controls and the (m)ActR-Fc-nLG3 dosed animals increased during the experiment. (m)ActR-Fc-nLG3 dosed animals increased more rapidly in BW and were on average 5.5% heavier than PBS control animals throughout the experiment. The increase in BW was statistically significant only at certain days of dosing, but not consistently throughout the dosing period (5 weeks) (S2A Fig). Previous publications have shown that myostatin inhibitors increase muscle strength [15, 19]. In order to determine if the (m)ActR-Fc-nLG3 compound induced a similar effect we measured grip strength during one week in either the 4th or the 5th week of dosing. The grip strength of (m)ActR-Fc-nLG3 mice (66.3 ± 3.4) was increased by 24% (P = 0.0148) compared to controls (53.7 ± 3.8). This corresponded with a slight increase in measured muscle weight (MW): the wet MW was increased on average by 9%. Only the weight increase of the triceps was statistically significant (P<0.05) (S2B Fig).

At first sight, the results were similar to what has previously been described for a myostatin inhibitor, but MWs and BWs were only very moderately increased [18, 19, 23], possibly because of poor tissue penetration of (m)ActR-Fc-nLG3. However, despite the diminished hypertrophy, there was a significant increase in grip strength as expected from a myostatin inhibitor [15, 19]. This intriguing observation led us to test the controls compounds (m)ActR-Fc and Fc-nLG3 and investigate our findings in more detail.

**Fig 1:**
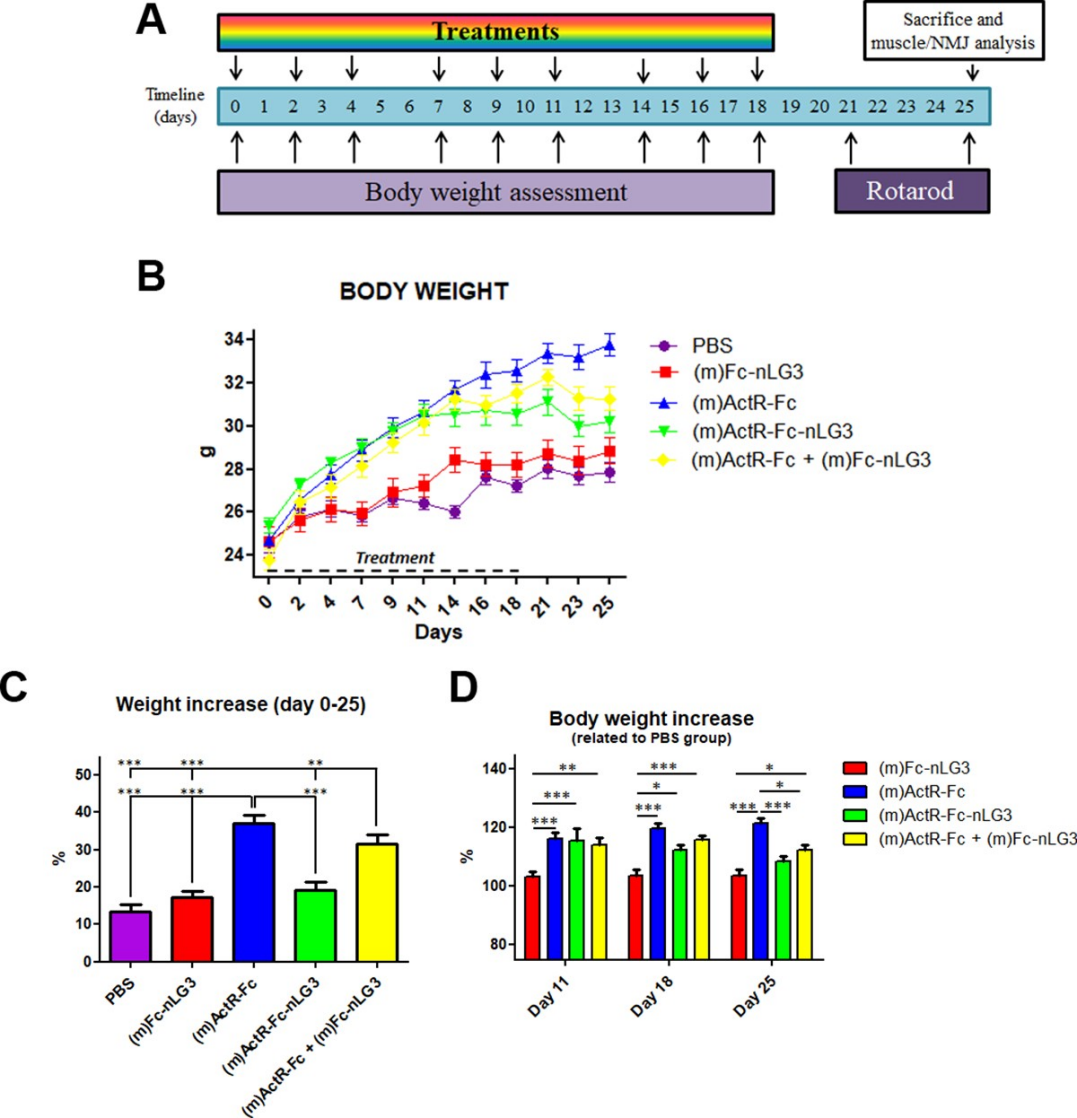
Evaluation of body weight. **(A)** Experimental timeline. **(B)** Weight increase displayed by PBS, (m)ActR-Fc, (m)Fc-nLG3, (m)ActR-Fc, (m)ActR-Fc-nLG3 and (m)ActR-Fc + (m)Fc-nLG3 treated groups. Two way ANOVA repeated measures: (m)ActR-Fc vs PBS, ***P < 0.001 from day 7 to day 21; (m)ActR-Fc + (m)Fc-nLG3 vs PBS, *P < 0.05 at day 7, **P < 0.01 at day 9, ***P < 0.001 from day 11 to day 21 (m)ActR-Fc-nLG3 vs PBS, *P < 0.05 at day 4, 23 and 25; ***P<0.001 from day 7 to day 21. **(C)** Weight increase between day 0 and day 25 expressed as a percentage; one way ANOVA, Bonferroni post hoc test **P < 0.01, ***P<0.001. **(D)** BW increase reached at day 11, day 18 and day 25, expressed by attributing to PBS group the value of 100% (two way ANOVA, Bonferroni post hoc test *P<0.05, **P < 0.01, ***P<0.001).

For this purpose, other nine-week-old animals were randomized on body weight (BW; average value 24.61 ± 0.48 g) and treated with the “single” constructs [i.e. (m)ActR-Fc and (m)Fc-nLG3] as well as with a mixture of both constructs [(m)ActR-Fc + (m)Fc-nLG3]. The results were compared to the “double” compound [(m)ActR-Fc-nLG3] and a PBS control group. The animals were treated between day 0 and day 18, and weighed at regular intervals throughout the experiment until day 25 (Fig 1A).

The BW increase of (m)Fc-nLG3 mice (17.22% at day 25) was not significantly different from that of the PBS group (13.34% at day 25) (Fig 1B and 1C). As expected, (m)ActR-Fc administration resulted in a steady and significant weight increase compared to PBS group (P<0.001 from day 7 to day 25; weight increase at day 25 is 36.95%). Also (m)ActR-Fc in the mixture with (m)Fc-nLG3 caused a highly significant BW increase of 31.43% at day 25 (P<0.05 at day 7, P<0.001 from day 11 to day 25). The BW increase induced by the “double” compound (m)ActR-Fc-nLG3 was similar to what observed in the previous experiment, and was only significantly different from the PBS group between day 7 and day 21 (P<0.001), but not on day 4, 23 and 25 (P>0.05). The average weight increase of this group on day 25 was 19.07%.

We have also calculated the BW increase at day 11, 18 and 25, by attributing a value of 100% to the PBS group, as shown in Fig 1D. Until day 11, the percent BW increase was similar among (m)ActR-Fc, (m)ActR-Fc-nLG3 and the mixture group, and statistically different compared to (m)Fc-nLG3, whose values remain constant during this time (until day 25). Then, after the treatment interruption (day 18), "(m)ActR-Fc + (m)Fc-nLG3” and (m)ActR-Fc-nLG3 groups showed a partial weight decline, whereas the values for the (m)ActR-Fc group remained unchanged until day 25 (Fig 1B and 1D). Interestingly, at day 25, (m)ActR-Fc-nLG3 was no longer statistically different from (m)Fc-nLG3.

### Rotarod test

Throughout the experiments the individual and social behavior of all mice appeared normal without unhealthy signs. Animals treated with the test compounds did not differ from the controls under all aspects. Food and water consumption also were identical/similar among groups.

**Fig 2:**
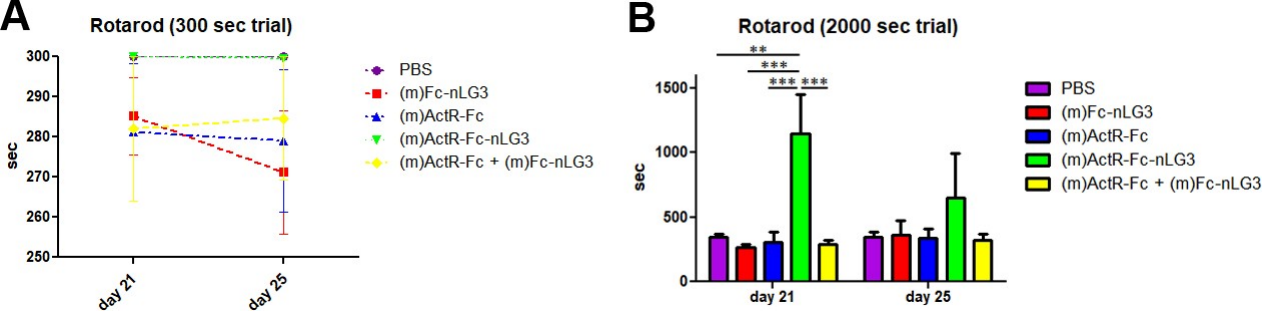
Motor performance on rotarod on day 21 and 25. **(A)** In the 300 sec trial no statistically significant differences among groups have been observed, whereas **(B)** in the 2000 sec trial (m)ActR-Fc-nLG3 mice demonstrated an unexpected resistance, in particular on day 21 (Two way ANOVA repeated measures Treatment: F_(4,20)_ = 3.32; p = 0.0306; Time: F_(1,20)_ = 1.83; p = 0.1911; Bonferroni post hoc test: (m)ActR-Fc-nLG3 vs PBS **P<0.01; (m)ActR-Fc-nLG3 vs (m)Fc-nLG3 / (m)ActR-Fc / (m)ActR-Fc + (m)Fc-nLG3 ***P<0.001).

During the dosing period, the animals were subjected to a rotarod test for 300 sec or 2000 sec, to evaluate motor performance. In the “300 sec trial”, we did not observe any significant differences among groups (Fig 2A).

More interestingly, in the “2000 sec trial” (m)ActR-Fc-nLG3 mice demonstrated an unexpected endurance: in the first trial they were able to remain on the rotating cylinder for 1149±299 sec, compared to 347±20.1 sec for PBS (P<0.01), 302±82.6 sec for (m)ActR-Fc (P<0.001), 292.8±28.8 sec for the mixture (m)ActR-Fc and (m)Fc-nLG3 (P<0.001), and 262.80±29.5 sec for (m)Fc-nLG3 (P<0.001). This result was repeated in a second “2000 sec trial”, performed 4 days later. In this session the other test groups showed similar performance to the first test (ranging from 320 and 360 sec), whereas the (m)ActR-Fc-nLG3 group doubled these results (651.8±339.1), confirming a remarkable increase in performance in the rotarod test (Fig 2B).

### Muscle analysis

**Fig 3:**
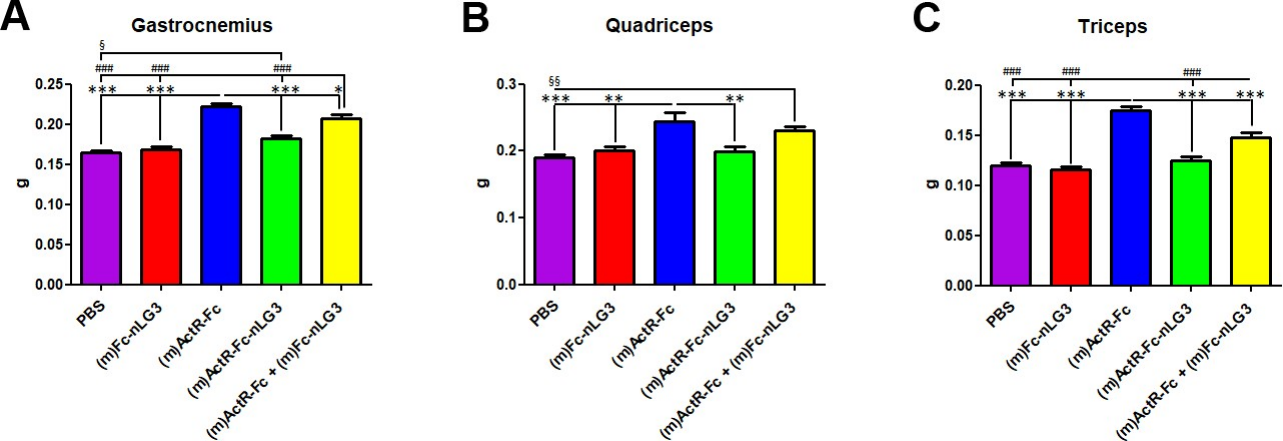
Wet weight (g) of gastrocnemius, quadriceps and triceps muscles. **(A)** Gastrocnemius: One-way ANOVA F_(4,45)_ = 46.02; p<0.0001; Bonferroni post hoc test (m)ActR-Fc vs PBS / (m)Fc-nLG3 / (m)ActR-Fc-nLG3 ***P<0.001; (m)ActR-Fc vs (m)ActR-Fc + (m)Fc-nLG3 *P<0.05; (m)ActR-Fc + (m)Fc-nLG3 vs PBS / (m)Fc-nLG3 / (m)ActR-Fc-nLG3 ###P<0.001; (m)ActR-Fc-nLG3 vs PBS §P<0.05; **(B)** Quadriceps: One-way ANOVA F_(4,45)_ = 9.313; p<0.0001; Bonferroni post hoc test (m)ActR-Fc vs PBS ***P<0.001, (m)ActR-Fc vs (m)Fc-nLG3 / (m)ActR-Fc-nLG3 **P<0.01; (m)ActR-Fc + (m)Fc-nLG3 vs PBS §§P<0.01; **(C)** Triceps: One-way ANOVA F_(4,45)_ = 43.98; p<0.0001; Bonferroni post hoc test (m)ActR-Fc vs PBS / (m)Fc-nLG3 / (m)ActR-Fc-nLG3 / (m)ActR-Fc + (m)Fc-nLG3 ***P<0.001, (m)ActR-Fc + (m)Fc-nLG3 vs PBS / (m)Fc-nLG3 / (m)ActR-Fc-nLG3 ###P<0.001.

Looking for an explanation for the observed effects on muscle strength and rotarod performance, we performed a histological analysis of the muscles. At the end of the observation period, the animals were sacrificed and muscles (gastrocnemius, quadriceps and triceps) isolated. In the gastrocnemius, the strongest increase in MW compared to PBS group was induced by (m)ActR-Fc compound (35.21%) alone and by the mixture [(m)ActR-Fc + (m)Fc-nLG3)] (25.74%). (m)ActR-Fc-nLG3 induced a more moderate MW gain of 10.87%. A similar trend was also seen for quadriceps and triceps. As expected, the “single” nLG3 construct did not affect muscle trophism. The overall data of the MW reflect, as expected the BW trend (Fig 3), as summarized in Table 2.

**Table 2:** Wet weight (g) of analyzed muscles (quadriceps, gastrocnemius, triceps). The values are represented as mean ± S.E.M.

	PBS	(m)Fc-nLG3	(m)ActR-Fc	(m)ActR-Fc-nLG3	(m)ActR-Fc + (m)Fc-nLG3
Quadriceps	0.190±0.016	0.201±0.017	0.244±0.040	0.200±0.023	0.231±0.017
Gastrocnemius	0.165±0.007	0.168±0.012	0.223±0.011	0.183±0.009	0.207±0.017
Triceps	0.119±0.012	0.116±0.009	0.175±0.013	0.125±0.011	0.148±0.014

The histological analyses of the gastrocnemius of PBS and (m)ActR-Fc-nLG3 groups revealed a significant increase in the mean fiber length as well as in the maximum Feret diameter of fibers (P≤0.05, unpaired T test) in the (m)ActR-Fc-nLG3 group, whereas the cross-sectional perimeter and the minimum Feret diameter did not significantly differ from the control group (Table 3).

**Table 3:** Morphological analysis of gastrocnemius of PBS and (m)ActR-Fc-nLG3 mice. Muscle perimeter, mean fiber length, maximum and minimum Feret diameter, and ratio nuclei/100 fibers are listed, and expressed as mean ± SEM (Student’s unpaired t-test; mean fiber length *P≤0.05; maximum Feret diameter #P<0.05).

	PBS	(m)ActR-Fc-nLG3
Total muscle perimeter (μm)	17961.04±272.97	19232.34±820.02
Mean fiber length (μm)	182.52±8.79	210.24±8.26 (*)
Max Feret diameter (μm)	68.10±3.35	79.63±2.91 (#)
Min Feret diameter (μm)	43.96±1.89	48.96±2.48
Ratio nuclei/100 fibers	1.89±0.19	2.13±0.15

We have also evaluated the number of nuclei per 100 muscle fibers. (m)ActR-Fc-nLG3 showed a slightly higher number of nuclei compared to the PBS group (2.13±0.15 versus 1.89±0.19), without reaching statistical significance.

### NMJ analysis

**Fig 4:**
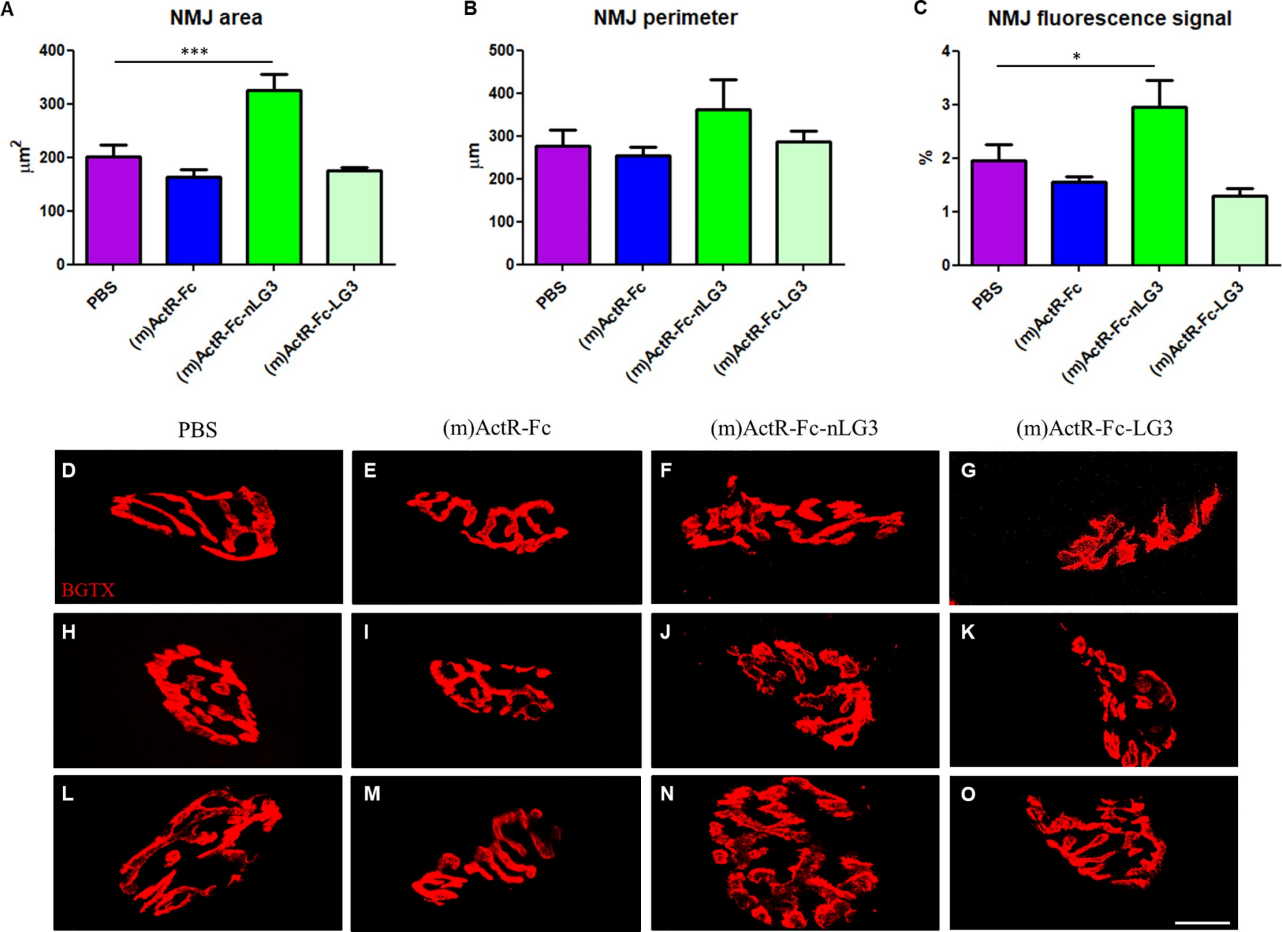
Morphometric analyses and confocal images of NMJs. **(A-B)** NMJ area and perimeter measured by ImageJ software (One-way ANOVA F_(3,36)_ = 14.00; p<0.0001; Dunnett's Multiple Comparison Test, PBS vs (m)ActR-Fc-nLG3 ***P<0.001); **(C)** Fluorescent signal intensity measured by Scion Image software (One-way ANOVA F_(3,31)_ = 7.791; p<0.005; Dunnett's Multiple Comparison Test, PBS vs (m)ActR-Fc-nLG3 *P<0.05). **(D-O)** Representative images of NMJs labeled by α-Bungarotoxin (BGTX, in red) for binding ACh receptors: **(D-H-I)** PBS-treated NMJs, **(E-I-M)** (m)ActR-Fc-treated NMJs, **(F-J-N)** (m)ActR-Fc-nLG3-NMJs, **(G-K-O)** (m)ActR-Fc-LG3-NMJs. Scale bar: 18 μm.

Muscle analysis of ActR-Fc-nLG3 treated animals did not provide a satisfactory explanation of the observed experimental rotarod results. It has been previously described that endurance training increases acetylcholine receptor quantity at neuromuscular junctions of adult rat skeletal muscle (26), so we decided to stain with fluorescent α-Bungarotoxin (BGTX) the NMJs of mice treated with PBS, (m)ActR-Fc, (m)ActR-Fc-nLG3 and (m)ActR-Fc-LG3 (Fig 4). Three representative NMJ pictures for each group are given in Fig 4. Further analysis showed that the area of NMJs of (m)ActR-Fc-nLG3 treated animals was significantly enlarged (P≤0.001, One-way ANOVA) compared to PBS treated animals (Fig 4A), indicating that more AChRs are present: conversely the other groups (namely, ActR-Fc and ActR-Fc-LG3 are not statistically different from PBS). The perimeter of (m)ActR-Fc-nLG3 NMJs was not statistically increased (Fig 4B), indicating growth of the NMJ area was achieved by thickening of the stained structures. Additionally in Fig 4C we quantified the BGTX fluorescent signal, confirming that only (m)ActR-Fc-nLG3 NMJs are significantly different from PBS group.

### *In vitro* AChR clustering

**Fig 5:**
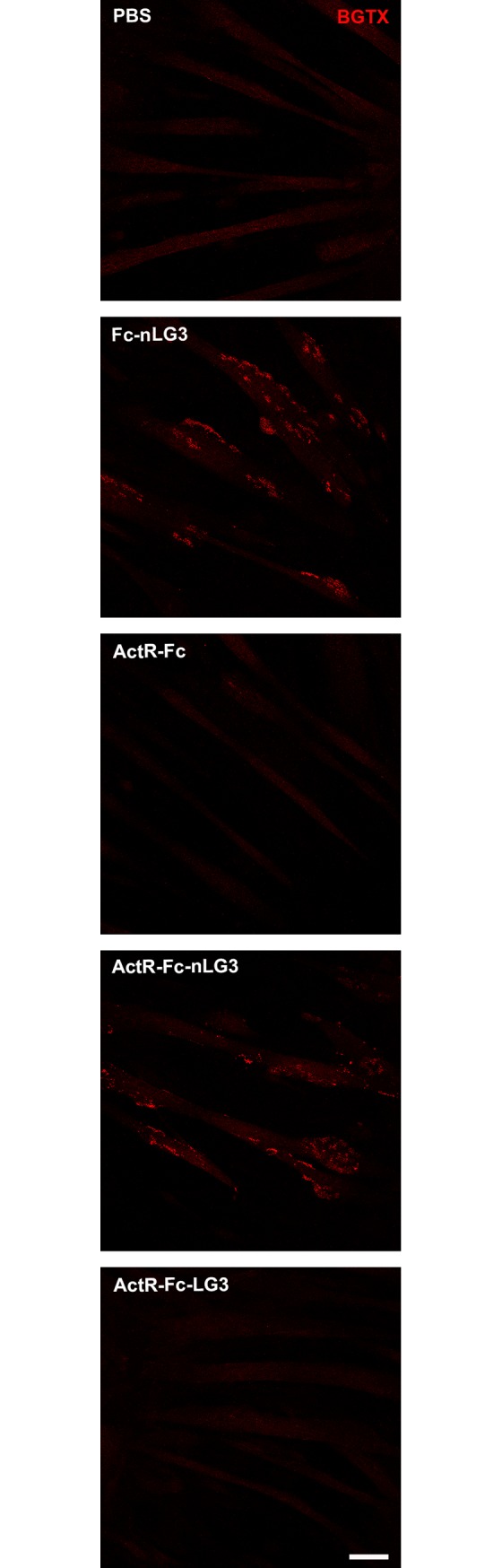
*In vitro* experiments. AChR clustering (see red α-Bungarotoxin-positive spots) induced by Fc-nLG3 and ActR-Fc-nLG3 in C2C12 skeletal myotubes. ActR-Fc and ActR-Fc-LG3 were not capable of inducing AChR clusters. Negative control is represented by PBS. Scale bar: 50 μm.

Agrin is a multidomain extracellular matrix protein. The most C-terminal domain of agrin is a laminin G domain (LG3) [12]. Neuronal agrin differs from non-neuronal agrin as it contains an 8 amino acid insertion in the C-terminal LG3 domain (nLG3). nLG3 is capable of phosphorylating the muscle-specific kinase (MuSK) by binding to the low-density lipoprotein receptor-related protein 4 (LRP4) receptor thereby inducing Ach receptor clusters on C2C12 cells, whereas non-neuronal agrin (LG3) cannot bind or signal via LRP4. In order to determine if signaling via the LRP4 receptor is important for the observed metabolic effects of ActR-Fc-nLG3, the human ActR-Fc was coupled to the human non-neuronal LG3 domain generating ActR-Fc-LG3. When we tested *in vitro* the proteins containing the neuronal agrin fragments [i.e., Fc-nLG3 and ActR-Fc-nLG3], we demonstrated the capability of promoting aggregation of the various components of the NMJs only at a concentration of 10 μM (Fig 5), which is a 1000-fold above the reported EC50 of the nLG3 protein (10 nM) alone [12]. At ten-fold lower concentrations clusters were hardly visible. As expected, ActR-Fc and ActR-Fc-LG3 were completely inactive in this assay (Fig 5). Since Fc-nLG3 and ActR-Fc-nLG3 were about equally active in inducing AChR clustering, it is likely that the Fc part makes the protein more soluble, thus less prone to binding to the wall of the myotube resulting in lower than expected efficacies. So, although ActR-Fc-nLG3 is active only at very high concentration *in vitro*, it does not mean that the nLG3 component is not capable of signaling via the LRP4 receptor *in vivo* at much lower concentration.

### Neuronal LG3 (nLG3) is required

We subsequently examined the effects of ActR-Fc-LG3 on mice using ActR-Fc-nLG3 and ActR-Fc as controls. If ActR-Fc-LG3 treated animals resemble ActR-Fc-nLG3 treated animals, it would mean that LRP4 binding does not play an important role. However, if ActR-Fc-LG3 treated animals resemble ActR-Fc treated animals, it means that signaling via LRP4 is important.

**Fig 6:**
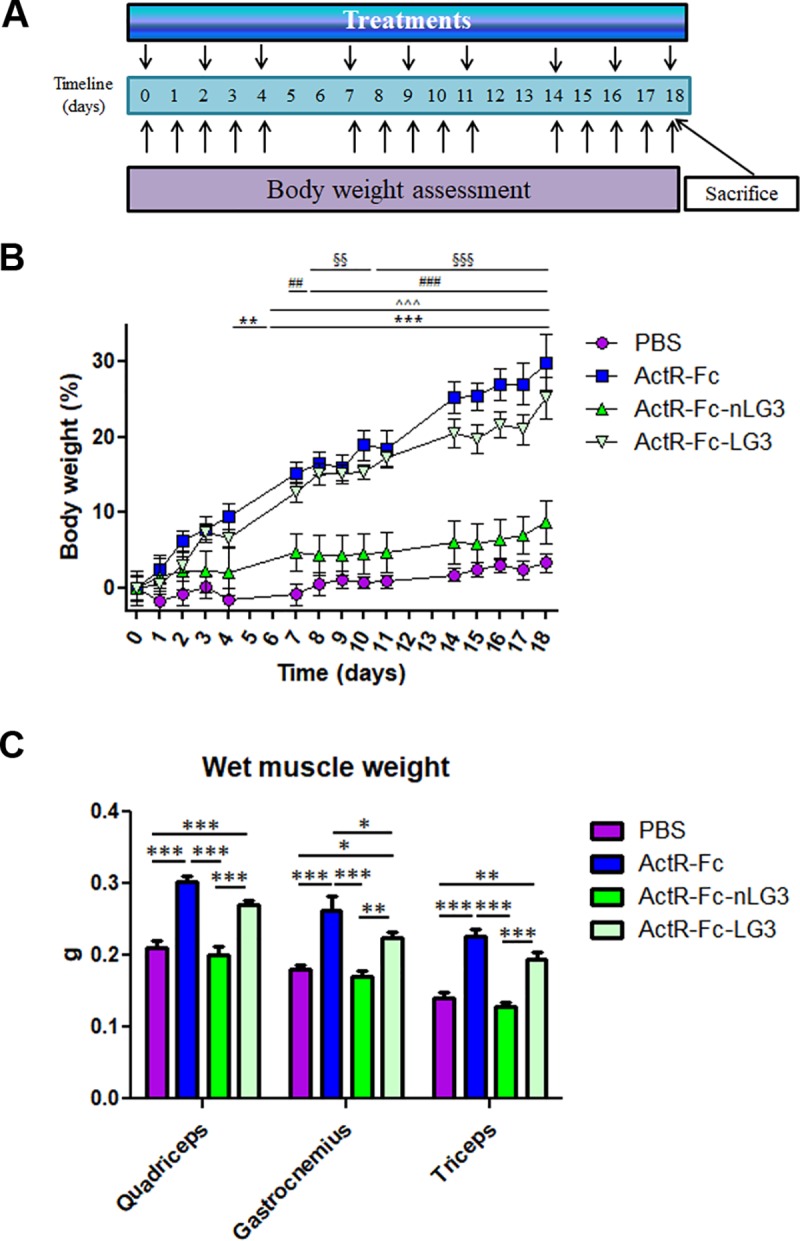
Effects of nLG3 and LG3 on body weight and muscle weight. **(A)** Experimental timeline. **(B)** Percent increase of BW (referred to PBS mice) over 3 weeks of treatment with ActR-Fc, ActR-Fc-nLG3, and ActR-Fc-LG3, dosed three times per week 10 mg/kg. Treatment: F_(3,140)_ 214.52; p<0.0001; Time: F_(14,240)_ = 29.55; p<0.0001; Bonferroni post hoc test: PBS vs ActR-Fc: P<0.01 at day 4, P<0.001 from day 7 to day 18; PBS vs ActR-Fc-LG3: P<0.001 from day 7 to day 18. ActR-Fc vs ActR-Fc-nLG3: P<0.01 at day 7 (##), P<0.001 from day 8 to day 18 (###); ActR-Fc-nLG3 vs ActR-Fc-LG3: P<0.01 from day 8 to day 10 (§§), P<0.001 from day 11 to day 18 (§§§). **(C)** Wet muscle weights of gastrocnemius, quadriceps and triceps muscles. Two way ANOVA repeated measures Treatment: F_(3,48)_ = 61.18; p<0.0001; Muscle: F_(2,48)_ = 53.34; p<0.0001; Bonferroni post hoc test: *P<0.05, **P<0.01, ***P < 0.001.

The treatment and body weight assessment schedule are illustrated in Fig 6A. After three weeks of dosing, ActR-Fc and ActR-Fc-LG3 induced a remarkable increase of BW compared to the PBS group (Fig 6B). The increase was particularly significant at the second and third weeks of dosage, with maximal increases reaching 30% for ActR-Fc and 25% for ActR-Fc-LG3 respectively. Most important, no significant differences have been observed during the whole period between ActR-Fc and ActR-Fc-LG3 treated animals. In addition, ActR-Fc-LG3 BWs were significantly higher than BWs of ActR-Fc-nLG3 treated animals from dosing day 8 until the end (Fig 6B).

After the animals were sacrificed, we evaluated the wet MW of the gastrocnemius, the quadriceps and the triceps (Table 4; Fig 6C). MW of all muscles was significantly increased in ActR-Fc-LG3 and ActR-Fc treated groups compared to PBS group. Conversely, ActR-Fc-nLG3 treated mice did not show significant differences in wet muscle weight when compared to control mice. These results show that ActR-Fc and ActR-Fc-LG3 are both capable of inducing strong muscle hypertrophy.

**Table 4:** Wet weight (g) of gastrocnemius, quadriceps and triceps. The values are represented as mean ± S.E.M. The statistical analysis is reported in Fig 4.

	PBS	ActR-Fc	ActR-Fc-nLG3	ActR-Fc-LG3
Quadriceps	0.210±0.010	0.302±0.008	0.201±0.011	0.271±0.006
Gastrocnemius	0.180±0.007	0.263±0.020	0.170±0.008	0.224±0.009
Triceps	0.140±0.009	0.226±0.010	0.128±0.006	0.194±0.010

## Discussion

Potentiation of human muscle performance is an important health factor when motor activity fails due to pathological conditions and/or aging hampering the well-being of millions of people worldwide as a major cause of morbidity and disability [28].

Several inhibitors of the myostatin pathway have been produced with the therapeutic aim of stimulating muscle growth and/or preventing muscle loss in human muscle disease [21, 29, 30]. All these compounds have been shown to increase muscle growth in clinical settings, however, no effect was detected in the 6 min walking distance test (6MWD). The failure of the myostatin inhibitor in clinical testing requiring sustained activity (such as the 6MWD test), in spite of the clearly increased muscle mass and potency, suggests that muscle hypertrophy *per se* is not sufficient to maintain muscle performance beyond momentary effort [21, 30].

In this study, we focused on combining neuromuscular effects with muscle hypertrophy, hypothesizing that it these might result in optimized performance. Thus, we constructed a novel protein by adding the fragment of agrin (nLG3) that is capable of signaling via LRP4 [24] to the anti-myostatin agent (ActR-Fc).

A previous study showed that administration of myostatin inhibitors causes a substantial increase in BW due to muscle mass increase [31]. We therefore verified the effects of the combined ActR-Fc-nLG3 and single compounds (ActR-Fc, Fc-nLG3) on mouse BW. Our experiments confirmed the BW increases by the known myostatin inhibitor compound ActR-Fc. This compound is very similar if not identical in sequence to Ramatercept [23]. The results obtained in this study duplicate those in several reports in the literature, describing a remarkable gain in muscle mass in several animal species by inhibition of myostatin [3]. The administration of the nLG3 agrin fragment alone (Fc-nLG3) did not produce statistically significant changes throughout the experiment. Unexpectedly, by adding the nLG3 agrin fragment to the activin receptor inhibitor, the increase in BW is significantly less prominent than with ActR-Fc alone (Fig 1). It appears that the addition of the agrin moiety stifled the hypertrophic process and this effect was caused by a reduction in muscle hypertrophy (Fig 3). The nLG3 containing compound (ActR-Fc-nLG3) caused a weight increase compared to PBS, but this effect was transient (Fig 1) and most importantly the BW increase was significantly lower than expected. Surprisingly, the two single compounds ActR-Fc and nLG3-Fc administered together did have the same effect on BW as ActR-Fc alone, meaning that the coupling of the two moieties is needed for the reduced BW increase.

We also verified if binding to the LRP4 receptor is important for this observed reduced BW increase effect. To this aim, we made the compound ActR-Fc-LG3, without the 8 amino acids involved in binding to the LRP4 receptor. LG3 linked to ActR-Fc (ActR-Fc-LG3) exerted a similar effect to ActR-Fc (Fig 6). This suggests that the observed BW and muscle weight changes are mediated by activation of the cascade of events triggered by binding to LRP4 and MuSK activation. In addition, it also shows that there is no steric hindrance of the ActR domain by the nLG3 domain.

A remarkable increase in endurance in the rotarod test was noted in animals treated with ActR-Fc-nLG3 (Fig 2). The 300 sec rotarod test did not differentiate various groups although the (m)ActR-Fc-nLG3 treated group reached the best performance of all groups on day 21. However, when the trial time was extended to 2000 sec, the (m)ActR-Fc-nLG3 treated mice outperformed all the other groups, demonstrating an exceptional endurance in the rotarod test. This performance declined seven days after treatment end, but time spent on the rotarod was still the longest of all the groups. This strongly indicates that to induce long-term sustaining muscle activity, the activin receptor inhibitor and the neuronal agrin fragment need to be present on one molecule, as no enhancement of the motor performance was observed when the parts of the molecules were administered separately. To the best of our knowledge, only one recent report [32] describes the successful enhancement of endurance in motor performance. There are, however, important differences to the current study. Firstly, they used transgenic (TG) mice overexpressing nicotinamide phosphoribosyl transferase in muscle, whereas ActR-Fc-nLG3 can be given as a drug. Secondly, the enhanced endurance was seen in the TG mice after nicotinamide supplementation in the diet which *per se* did not induce the same effect in the wild types. Lastly, the endurance effect was obtained only after repetitive exercise. Notably, in our experiments the endurance on the rotarod was seen both at the first trial as well as after repetitive trials.

The observed myogenic findings are limited and probably cannot explain the surprising endurance effect, so we also looked at NMJ changes. In order to determine if ActR-Fc-nLG3 was able to induce postsynaptic changes on the NMJ, α-Bungarotoxin (BGTX) staining was performed on muscle tissue. Representative images of PBS, ActR-Fc-nLG3, ActR-Fc and ActR-Fc-LG3 samples are shown in Fig 4D–4O. The measurement of the surface confirmed a significantly increased NMJ surface area for ActR-Fc-nLG3 animals compared to PBS (Fig 4A). Similarly, performing a densitometric analysis of AchRs, we observed a significantly higher BGTX fluorescent signal for ActR-Fc-nLG3 animals (Fig 4C). These findings could provide an explanation for the remarkably increased rotarod performance in ActR-Fc-nLG3-treated mice reached without training.

It has been demonstrated that the NMJ has the capacity to undergo significant morphological changes as a result of increased exercise. For example, endurance training has been shown to increase the area of Ach receptors and the number of Ach receptors [33]. If training is capable of inducing these adaptations, it is feasible that treatment with ActR-Fc-nLG3, which induces similar changes to the NMJ, leads to an increased endurance.

LRP4, the receptor for neuronal agrin, acts as the functional step to transduce neuronal agrin activity. It is possible that ActR-Fc-nLG3 can bind outside the NMJ to the LRP4 receptor and enters the NMJ via lateral movement of the compound-LRP4 complex. Within the NMJ, ActR-Fc-nLG3 can form a complex with MuSK leading to auto-activation of this receptor and increased signaling of the corresponding pathway.

**Fig 7:**
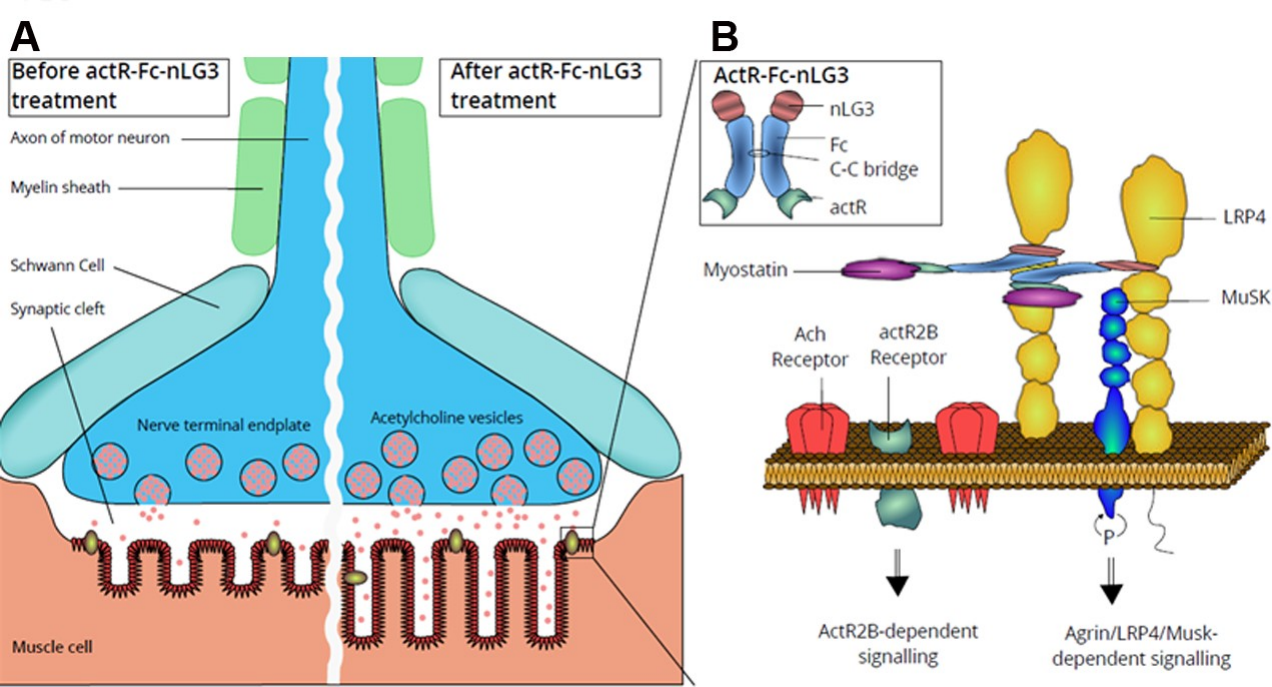
**Illustration of the NMJ (A, left panel) and the effects caused on it by ActR-Fc-nLG3 (A, right panel).** Schematic drawing of a presynaptic motor nerve terminal. ACh vesicles fuse with the terminal membrane releasing the neurotransmitter in the synaptic cleft which subsequently binds to the ACh receptor. **(A, right panel)** Postsynaptic changes potentially induced by ActR-Fc-nLG3 include enfolded junctional folds of the motor endplate amplifying the surface area for ACh receptors. **(B)** Schematic illustration of the hypothetical mechanism of action of ActR-Fc-nLG3. A schematic drawing of an ActR-Fc-nLG3 molecule is given in B (insert). The agrin-Lrp4-MuSK complex is considered essential for the formation and maintenance of the NMJ. ActR-Fc-nLG3 probably binds with its neural LG3 component to Lrp4 potentially activating the phosphorylation of muscle-specific tyrosine kinase receptor. ActR-Fc-nLG3 could also lead to myostatin inhibition within the synaptic cleft.

Signaling within the NMJ, however, does not only involve MuSK: myostatin and myostatin-like proteins regulate synaptic function and neuronal morphology [34] and GDF11 levels/expression increase with age [35]. In the case of ActR-Fc-nLG3, the soluble Act receptor is transported into the synaptic cleft because it is attached to the nLG3 protein. Within the synaptic cleft, ActR-Fc-nLG3 can bind and inactivate myostatin and/or activin excreted into the NMJ by the muscle fiber (Figs 1–7). Emerging knowledge of the effects of myostatin and myostatin-related proteins has recently shown in Drosophila that a homolog of myostatin and GDF11 regulates not only BW and muscle size, but also inhibits neuromuscular synapse strength and composition [34].

In this respect it is worth noting that agrin has a number of Follistatin-like domains that are located at its N-terminus [12]. Follistatin is known to bind and inhibit myostatin and other members of the TGFbeta family [3]. The Follistatin-like domains of agrin are known to bind BMP2, BMP4, and members of the TGFbeta family. It is also well-known that proteases regulate the activity of Agrin [36, 37]. Proteolysis separates the N- and C-terminus of agrin, and thus splits the Follistatin-like domains from the nLG3 domain. In the example of ActR-Fc-nLG3 the ActR domain is separated from the nLG3 domain. This could mean that ActR-Fc-nLG3 represents a novel form of full-length Agrin.

We are planning further studies to investigate reporter genes that are up- or down-regulated after administration of our compounds as the first step toward entangling the putative interferences among these multiple pathways. In parallel, to confirm the pivotal role of NMJs in improving endurance, we plan to investigate if the same increment of enduring motor performance can also be observed in aged mice, where weakening and fragmentation of the NMJ is known to occur [38].

## Supporting information

S1 Dataset Raw data of in vivo and ex vivo experiments. (PDF)

S1 Fig Assessment of the molecular weight of the synthesized compounds. SDS-PAGE results for mouse **(A)** and human **(B)** derivatives, stained with Coomassie Blue. **(C)** Table showing the calculated volume (μl) for each human and mouse derivatives, to load 1μg of each compound on the SDS-polyacrylamide gel.
(TIF)

S2 Fig Preliminary observations after dosing of (m)ActR-Fc-nLG3. **(A)** Throughout the dosing period of 5 weeks, the body weight of (m)ActR-Fc-nLG3 animals moderately increased compared to PBS-mice; **(B)** similarly, the muscle weight was slightly affected by the treatment (only the triceps show a significant MW increased compared to control animals; Student’s unpaired t-test, *P<0.05).
(TIF)
