# Supplementary material for: A new protein curbs the hypertrophic effect of myostatin inhibition, adding remarkable endurance to motor performance in mice
Source: PLoS One. 2020 Mar 11;15(3):e0228653. doi: 10.1371/journal.pone.0228653 (PMC7065788; doi:10.1371/journal.pone.0228653)
Supplement: S1 Dataset — (PDF) [file pone.0228653.s001.pdf]

Evaluation of body weight (g)

	PBS	(m)Fc-nLG3	(m)ActR-Fc	(m)ActR-Fc-nLG3	(m)ActR-Fc + (m)Fc-nLG3
day 0	24.8	27.4	25.5	26.0	23.0
	24.4	24.2	24.7	25.4	25.5
	23.0	23.3	24.9	25.5	24.1
	25.8	23.5	25.0	24.1	22.8
	24.9	24.7	23.3	25.9	23.6
day 2	25.7	27.5	27.3	27.2	25.5
	25.8	25.7	26.4	28.0	28.4
	25.1	24.5	26.7	27.1	26.7
	27.0	24.6	27.4	26.3	26.0
	25.3	25.9	25.1	27.8	25.7
day 4	25.7	28.2	28.5	28.6	26.5
	26.1	26.1	27.6	28.2	29.3
	25.9	24.9	27.7	28.1	27.2
	27.6	25.0	28.8	27.8	26.5
	25.5	26.5	26.3	29.0	26.5
day 7	25.1	27.7	29.5	28.9	27.7
	25.8	26.1	28.3	28.7	30.1
	26.2	24.6	28.6	28.6	28.2
	27.0	25.1	30.4	28.5	27.6
	25.3	26.3	27.6	30.5	27.2
day 9	25.9	29.2	30.6	29.3	29.2
	26.6	26.8	29.1	29.7	31.0
	26.5	26.0	29.9	29.0	29.0
	27.8	25.5	31.2	29.2	28.9
	26.6	27.1	28.9	31.5	28.2
day 11	26.0	29.0	31.5	30.0	30.3
	26.4	27.0	29.5	30.2	32.3
	26.3	26.3	30.9	29.8	30.2
	27.5	26.1	32.1	30.3	29.2
	25.9	27.8	29.5	32.4	28.9
day 14	25.5	30.3	32.3	30.2	31.1
	26.2	28.0	30.6	30.1	32.9
	26.0	27.2	32.0	29.7	31.7
	27.1	27.5	32.8	30.0	30.5
	25.4	29.1	31.0	32.8	30.0
day 16	27.0	30.0	33.3	30.4	31.1
	27.1	28.4	31.0	30.2	32.6
	27.6	27.0	33.0	29.2	31.1
	28.7	27.0	33.8	30.6	30.1
	27.7	28.8	30.9	33.4	29.9
day 18	26.7	30.2	32.7	30.6	31.5
	27.3	28.1	31.1	29.9	33.1
	27.1	26.9	33.3	29.4	31.5
	28.4	27.3	34.1	30.6	30.9
	26.7	28.6	31.7	32.4	30.6
day 21	27.1	30.8	33.8	31.5	32.4
	28.3	29.1	31.9	30.8	33.5
	27.9	27.2	34.2	29.7	32.3
	29.5	27.5	34.3	30.4	32.0
	27.3	29.0	32.8	33.2	31.2
day 23	27.0	30.3	33.5	30.2	31.8
	28.2	28.6	31.4	29.6	33.2
	26.8	26.9	33.9	29.0	30.9
	29.1	27.0	34.5	29.4	30.6
	27.4	29.2	32.7	31.8	30.0
day 25	27.2	30.7	33.5	30.3	32.1
	27.5	29.0	32.6	29.7	32.9
	27.5	27.3	34.6	28.9	30.7
	29.6	27.8	35.3	30.1	30.7
	27.5	29.5	33.0	32.1	30.0

% Weight increase (day 0- 25)

PBS	(m)Fc-nLG3	(m)ActR-Fc	(m)ActR-Fc-nLG3	(m)ActR-Fc + (m)Fc-nLG3
9.68	12.04	31.37	16.54	39.57
12.7	19.83	31.98	16.93	29.02
19.57	17.17	38.96	13.33	27.39
14.73	18.3	41.2	24.9	34.65
10.44	19.43	41.63	23.94	27.12

% Body weight increase (related to PBS group)

	(m)Fc-nLG3	(m)ActR-Fc	(m)ActR-Fc-nLG3	(m)ActR-Fc + (m)Fc-nLG3
Day 11	109.77	119.23	113.55	114.69
	102.20	111.66	114.31	122.26
	99.55	116.96	112.79	114.31
	98.79	121.50	114.69	110.52
	105.22	111.66	122.63	109.39
Day 18	110.87	120.04	112.33	115.64
	103.16	114.17	109.77	121.51
	98.75	122.25	107.93	115.64
	100.22	125.18	112.33	113.44
	104.99	116.37	118.94	112.33
Day 25	110.19	120.24	108.76	115.22
	104.09	117.01	106.60	118.09
	97.99	124.19	103.73	110.19
	99.78	126.70	108.04	110.19
	105.89	118.45	115.22	107.68

Rotarod (300 sec trial)

	PBS					(m)Fc-nLG3					(m)ActR-Fc					(m)ActR-Fc-nLG3					(m)ActR-Fc + (m)Fc-nLG3				
day 21	300	300	300	300	300	274	300	300	300	252	213	300	300	300	293	300	300	300	300	300	300	300	300	300	210
day 25	300	300	300	300	300	225	287	300	300	244	209	300	300	286	300	298	300	300	300	300	300	300	300	300	223

Rotarod (2000 sec trial)

	PBS					(m)Fc-nLG3					(m)ActR-Fc					(m)ActR-Fc-nLG3					(m)ActR-Fc + (m)Fc-nLG3				
day 21	396	372	280	360	327	273	283	185	356	217	151	279	622	220	238	318	2000	909	877	1641	382	247	303	316	216
day 25	328	503	300	312	270	282	281	303	794	140	145	226	444	299	567	204	2000	355	274	426	294	288	460	377	174

Wet weight (g) of gastrocnemius

PBS	(m)Fc-nLG3	(m)ActR-Fc	(m)ActR-Fc-nLG3	(m)ActR-Fc + (m)Fc-nLG3
0.165	0.173	0.225	0.189	0.223
0.158	0.162	0.196	0.173	0.224
0.167	0.144	0.233	0.177	0.194
0.173	0.161	0.222	0.171	0.184
0.158	0.170	0.213	0.192	0.196
0.170	0.188	0.228	0.186	0.218
0.169	0.164	0.225	0.181	0.236
0.153	0.173	0.222	0.179	0.200
0.172	0.165	0.234	0.179	0.199
0.162	0.184	0.229	0.199	0.197

Wet weight (g) of quadriceps

PBS	(m)Fc-nLG3	(m)ActR-Fc	(m)ActR-Fc-nLG3	(m)ActR-Fc + (m)Fc-nLG3
0.198	0.228	0.252	0.211	0.257
0.198	0.194	0.262	0.209	0.224
0.186	0.181	0.190	0.158	0.219
0.196	0.217	0.294	0.203	0.239
0.180	0.201	0.176	0.226	0.206
0.205	0.212	0.246	0.207	0.259
0.197	0.174	0.243	0.179	0.225
0.175	0.190	0.282	0.200	0.230
0.207	0.197	0.217	0.175	0.235
0.156	0.213	0.282	0.229	0.213

Wet weight (g) of triceps

PBS	(m)Fc-nLG3	(m)ActR-Fc	(m)ActR-Fc-nLG3	(m)ActR-Fc + (m)Fc-nLG3
0.127	0.129	0.169	0.111	0.160
0.124	0.114	0.181	0.126	0.154
0.115	0.115	0.191	0.116	0.145
0.141	0.128	0.188	0.133	0.137
0.111	0.108	0.183	0.126	0.132
0.124	0.116	0.158	0.122	0.179
0.122	0.123	0.151	0.140	0.142
0.100	0.102	0.170	0.108	0.154
0.123	0.115	0.185	0.135	0.140
0.107	0.110	0.172	0.132	0.136

Total muscle perimeter (μm)

PBS	(m)ActR-Fc-nLG3
18125.7	18367.2
17416.9	18495.9
17379.0	21598.8
18869.6	20619.8
18014.0	17080.0

Mean fiber length (μm)

PBS	(m)ActR-Fc-nLG3
210.5	203.6
187.1	238.3
155.6	214.2
180.7	207.4
178.7	187.7

Max Feret diameter (μm)

PBS	(m)ActR-Fc-nLG3
78.585	74.982
70.162	89.387
57.768	80.919
67.806	80.339
66.180	72.506

Min Feret diameter (μm)

PBS	(m)ActR-Fc-nLG3
50.309	48.538
43.712	56.588
38.384	51.247
43.811	46.898
43.599	41.541

Ratio nuclei/100 fibers

PBS	(m)ActR-Fc-nLG3
2.45	2.19
2.01	2.02
1.61	2.70
2.03	1.85
1.35	1.90

NMJ Area (μm2)

PBS	(m)ActR-Fc	(m)ActR-Fc-nLG3	(m)ActR-Fc-LG3
233.4	142.7	323.1	188.3
235.8	195.0	336.2	153.1
245.6	157.5	290.9	142.3
108.3	229.5	346.0	205.3
273.4	207.5	301.8	168.1
144.0	207.2	521.8	167.2
172.0	132.6	422.3	146.0
107.7	131.0	291.1	190.5
207.9	117.9	192.6	181.8
294.4	106.2	219.5	203.4

NMJ Perimeter (μm)

PBS	(m)ActR-Fc	(m)ActR-Fc-nLG3	(m)ActR-Fc-LG3
314.7	245.4	292.2	199.2
273.6	276.8	353.6	277.9
331.3	316.4	399.1	174.7
125.2	260.1	212.6	340.1
463.5	378.3	251.4	250.1
165.2	252.7	889.2	241.5
190.3	220.4	573.4	251.6
134.9	245.3	300.2	352.6
351.4	176.8	140.5	386.7
413.1	175.1	197.2	399.7

NMJ Fluorescence signal (%)

PBS	(m)ActR-Fc	(m)ActR-Fc-nLG3	(m)ActR-Fc-LG3
2.80	1.38	3.01	1.96
1.06	1.29	3.30	1.77
1.48	1.63	3.09	0.92
1.78	1.21	5.38	0.83
1.16	1.17	2.37	1.16
2.82	1.62	1.05	1.07
2.58	2.43	2.52	0.83
	1.87		1.34
	1.72		2.05
	1.32		0.94
	1.35		

Effects of nLG3 and LG3 on body weight (g)

	Baseline	Day 0	Day 1	Day 2	Day 3	Day 4	Day 7	Day 8	Day 9	Day 10	Day 11	Day 14	Day 15	Day 16	Day 17	Day 18
	24.1	24.8	24.4	24.7	24.6	24.6	25.1	25.3	25.2	25.2	25.1	25.4	25.4	25.8	25.5	25.7
	23.4	24.0	23.7	23.8	24.8	24.1	24.0	24.8	25.2	25.6	25.7	25.6	26.1	26.2	26.8	26.8
PBS	24.1	25.2	24.5	24.8	24.6	24.1	24.4	24.5	24.7	24.8	24.8	25.0	25.3	25.4	25.0	25.1
	25.2	25.7	25.1	25.4	25.6	25.1	25.3	25.3	25.7	25.3	25.3	25.7	25.7	25.6	25.4	25.8
	25.8	26.2	26.1	26.3	26.5	26.1	26.0	26.7	26.5	26.0	26.1	26.4	26.5	26.6	26.2	26.7
	25.8	25.7	26.2	26.8	27.1	27.5	29.1	29.8	29.4	30.4	31.3	32.1	32.0	32.1	32.4	33.6
	24.3	24.8	24.7	26.0	26.8	26.9	29.3	29.1	29.1	29.6	29.3	31.7	31.6	32.6	32.6	33.2
ActR-Fc	25.8	26.1	26.9	27.6	28.4	28.9	29.6	30.1	30.2	31.2	30.5	32.4	32.4	32.9	33.4	34.8
	24.8	24.3	25.7	26.4	26.5	27.0	27.9	28.3	28.3	28.6	28.5	30.2	30.5	30.6	30.2	30.5
	23.3	23.9	24.4	25.7	25.8	26.4	27.9	28.1	27.8	28.6	28.1	29.8	30.0	30.3	29.9	30.0
	23.5	23.1	23.5	23.6	23.6	23.6	24.3	24.2	24.1	24.3	24.4	24.7	24.5	24.6	24.8	24.7
	26.2	26.3	27.0	27.5	27.0	27.6	27.8	27.9	28.0	27.9	27.9	28.5	28.0	28.0	28.0	28.5
ActR-Fc-nLG3	25.2	25.7	25.9	25.8	26.6	26.6	26.6	26.7	26.4	26.9	26.9	26.9	27.3	27.2	27.6	28.0
	24.3	24.7	25.0	25.1	25.7	25.2	26.2	25.9	26.2	26.0	26.2	26.8	26.6	27.1	26.9	27.7
	24.2	24.4	24.7	25.0	24.0	23.8	25.1	24.8	25.0	24.7	24.7	24.8	25.2	25.4	25.5	26.1
	25.0	25.6	25.8	26.6	27.8	27.6	28.6	29.8	29.6	29.4	29.6	30.9	30.4	30.8	30.9	31.3
	23.7	24.8	24.5	25.2	26.4	26.2	27.2	27.9	27.9	28.2	28.7	28.7	28.5	29.1	28.7	29.3
ActR-Fc-LG3	25.3	25.8	26.2	26.5	27.5	27.7	29.2	29.8	29.6	29.5	30.0	30.5	30.3	31.1	30.5	31.6
	25.0	25.2	25.4	26.2	27.2	26.9	28.8	29.0	29.4	29.4	30.2	31.5	31.2	31.4	31.8	33.1
	24.8	25.1	25.4	25.9	27.0	26.6	28.8	29.1	29.1	29.3	29.7	30.8	31.0	31.4	31.1	33.1

Effects of nLG3 and LG3 on muscle weight (g)

Quadriceps

PBS	ActR-Fc	ActR-Fc-nLG3	ActR-Fc-LG3
0.213	0.315	0.210	0.277
0.218	0.336	0.178	0.266
0.193	0.303	0.183	0.269
0.218	0.294	0.248	0.295
0.221	0.310	0.203	0.293
0.242	0.295	0.194	0.258
0.195	0.290	0.182	0.248
0.157	0.318	0.167	0.266
0.222	0.283	0.225	0.265
0.219	0.279	0.219	0.274

Gastrocnemius

PBS	ActR-Fc	ActR-Fc-nLG3	ActR-Fc-LG3
0.180	0.278	0.199	0.214
0.191	0.313	0.168	0.210
0.177	0.309	0.189	0.228
0.181	0.204	0.168	0.210
0.193	0.193	0.160	0.254
0.204	0.250	0.169	0.233
0.153	0.318	0.168	0.238
0.167	0.280	0.173	0.190
0.168	0.222	0.165	0.218
0.181	0.258		0.247

Triceps

PBS	ActR-Fc	ActR-Fc-nLG3	ActR-Fc-LG3
0.180	0.218	0.123	0.172
0.143	0.240	0.157	0.177
0.113	0.233	0.128	0.207
0.120	0.209	0.130	0.200
0.162	0.212	0.121	0.216
0.126	0.230	0.131	0.173
0.142	0.281	0.124	0.197
0.143	0.225	0.140	0.201
0.126	0.195	0.115	0.207
0.143	0.214		0.192

Preliminary observations after dosing of (m)ActR-Fc-nLG3

Body weight (g)

	Day 1	Day 3	Day 5	Day 8	Day 10	Day 12	Day 15	Day 17	Day 19	Day 22	Day 24	Day 26	Day 29	Day 31	Day 33
PBS	26.6	26.6	26.7	27.0	27.0	27.4	28.0	28.6	29.0	29.0	28.9	29.3	29.4	28.9	29.3
	31.4	31.4	31.7	32.8	32.2	33.0	33.5	33.6	34.0	33.0	32.8	32.6	33.4	32.5	33.0
	28.7	29.0	29.3	28.8	28.9	28.4	29.3	30.1	29.3	29.5	29.7	29.4	30.3	26.9	30.4
	27.1	27.7	27.7	29.0	30.1	29.3	30.5	31.3	30.8	30.9	30.6	30.9	31.1	27.4	30.5
	29.2	29.8	30.1	29.8	29.9	30.1	30.9	30.4	30.5	30.2	30.8	30.9	31.2	27.4	30.7
	28.8	29.1	29.6	30.1	30.6	30.9	31.1	31.9	31.1	31.2	31.1	31.6	31.1	31.0	29.6
	31.0	31.4	32.0	32.0	31.4	31.8	32.3	32.0	31.5	32.1	32.4	32.4	32.7	32.4	30.9
	27.5	28.1	29.2	28.6	29.0	28.7	29.7	29.1	29.6	28.8	29.5	29.4	29.3	28.9	27.7
	28.8	28.9	29.1	28.8	29.0	29.2	29.9	30.5	31.0	31.2	31.5	30.9	31.3	30.1	29.6
	26.4	27.9	27.3	28.6	28.9	29.2	31.0	30.4	31.2	31.1	30.9	30.5	31.4	30.2	30.1
(m)ActR-Fc-nLG3	26.7	26.9	28.0	29.0	29.3	29.3	29.2	29.6	29.6	29.4	29.2	30.3	30.6	29.1	29.2
	29.6	30.6	31.0	32.0	31.3	31.6	31.0	31.5	32.2	31.7	31.9	32.7	32.8	31.7	32.1
	29.2	29.9	31.6	31.8	32.2	31.9	32.0	33.1	32.7	32.4	32.3	32.7	32.8	31.9	31.2
	30.0	30.5	31.4	32.0	32.0	31.8	32.0	31.7	31.5	31.5	31.7	32.7	32.0	28.1	31.5
	28.2	29.1	30.1	30.5	30.5	30.5	30.4	30.4	30.0	30.5	30.1	30.7	31.7	28.1	30.3
	30.2	31.4	31.8	31.8	32.3	32.0	32.3	32.3	32.9	32.1	32.9	33.8	34.1	33.0	31.4
	28.8	30.5	30.7	31.0	30.6	31.2	31.1	31.3	31.8	31.6	31.8	32.5	32.2	31.0	30.9
	31.7	33.2	33.9	34.6	34.4	35.4	34.6	35.3	35.2	35.0	35.3	35.0	35.8	34.7	34.1
	28.4	30.5	31.1	31.4	31.3	31.8	31.4	31.5	31.8	32.1	32.1	32.7	32.7	31.7	31.5
	29.3	30.3	31.1	32.3	32.4	33.0	33.3	33.7	33.9	34.4	34.2	34.5	35.2	33.6	33.4

Muscle weight (g)

Gastrocnemius		Quadriceps		Triceps	
PBS	(m)ActR-Fc-nLG3	PBS	(m)ActR-Fc-nLG3	PBS	(m)ActR-Fc-nLG3
0.164	0.188	0.212	0.173	0.116	0.120
0.184	0.185	0.209	0.239	0.119	0.136
0.157	0.199	0.188	0.203	0.109	0.144
0.187	0.188	0.184	0.200	0.124	0.135
0.174	0.160	0.123	0.198	0.107	0.142
0.206	0.198	0.197	0.224	0.125	0.161
0.191	0.214	0.194	0.230	0.096	0.164
0.174	0.192	0.173	0.217	0.124	0.135
0.189	0.184	0.195	0.180	0.123	0.144
0.204	0.197	0.204	0.215	0.128	0.143
0.155	0.137	0.131	0.126	0.092	0.118
0.180	0.166	0.185	0.130	0.134	0.165
0.177	0.181	0.154	0.149	0.135	0.156
0.204	0.206	0.190	0.250	0.164	0.151
0.155	0.203	0.141	0.155	0.132	0.109
0.207	0.210	0.201	0.187	0.131	0.162
0.198	0.198	0.156	0.225	0.143	0.176
0.171	0.199	0.159	0.149	0.127	0.148
0.184	0.195	0.166	0.130	0.130	0.136
0.200	0.210	0.205	0.207	0.118	0.165
