# Supplementary figures and images for: A new protein curbs the hypertrophic effect of myostatin inhibition, adding remarkable endurance to motor performance in mice
Source: PLoS One. 2020 Mar 11;15(3):e0228653. doi: 10.1371/journal.pone.0228653 (PMC7065788; doi:10.1371/journal.pone.0228653)

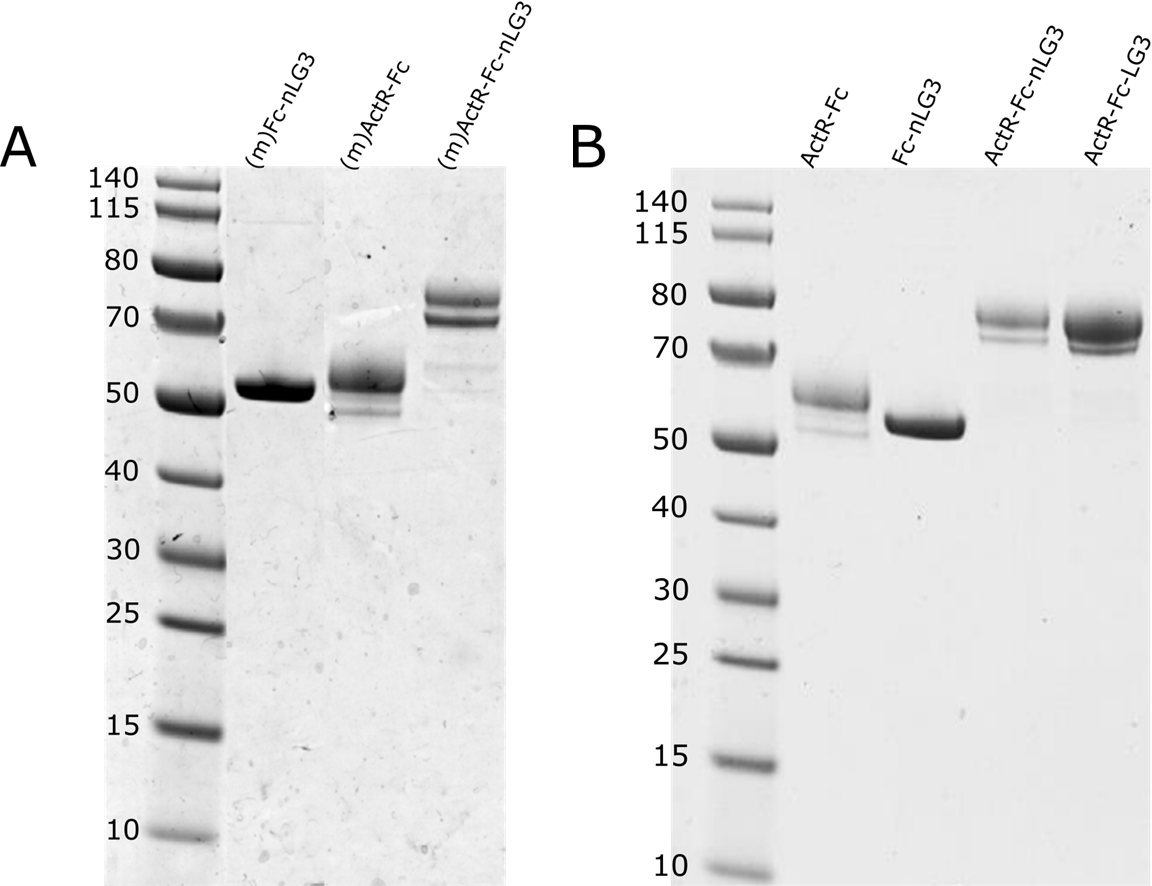

Supplement: S1 Fig — SDS-PAGE results for mouse (A) and human (B) derivatives, stained with Coomassie Blue. (C) Table showing the calculated volume (μl) for each human and mouse derivatives, to load 1μg of each compound on the SDS-polyacrylamide gel. (TIF) [file pone.0228653.s002.tif]

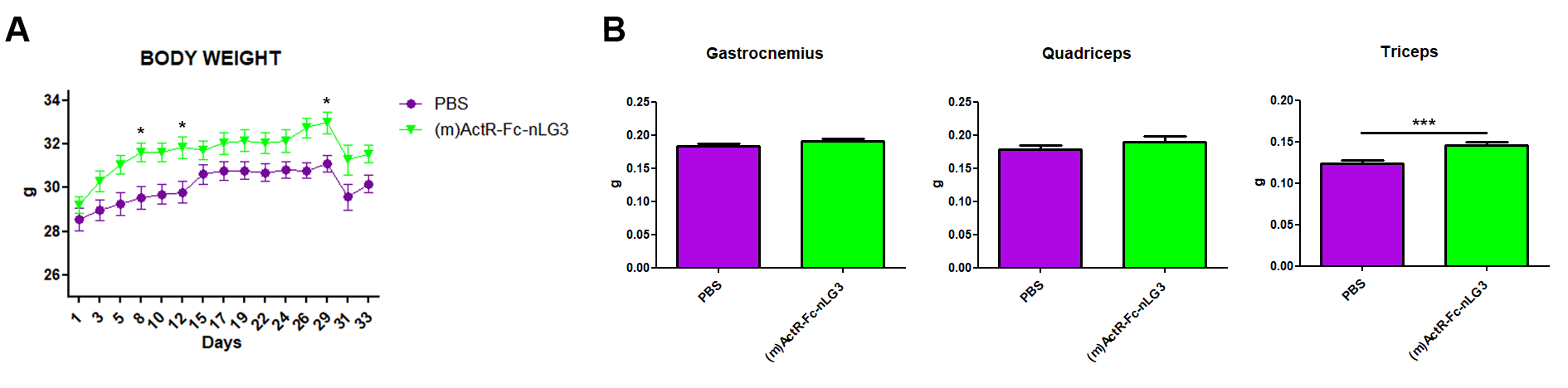

Supplement: S2 Fig — (A) Throughout the dosing period of 5 weeks, the body weight of (m)ActR-Fc-nLG3 animals moderately increased compared to PBS-mice; (B) similarly, the muscle weight was slightly affected by the treatment (only the triceps show a significant MW increased compared to control animals; Student’s unpaired t-test, *P<0.05). (TIF) [file pone.0228653.s003.tif]
